# *TIMP1* and *DPP4* Promote Tumor Progression by Regulating Lactate Metabolism in Papillary Thyroid Carcinoma

**DOI:** 10.3390/cancers18081264

**Published:** 2026-04-16

**Authors:** ShiJi Mu, Jin Xue, Fada Xia, Xiwu Ouyang, Guode Fu, Ruotong Gui, Haihong Wang, Ning Bai

**Affiliations:** 1 The Second Clinical College, Xinjiang Medical University, Urumqi 830018, China; 18355426569@163.com (S.M.); fgd202603@163.com (G.F.); 17381366793@163.com (R.G.); whh00826@163.com (H.W.); 2 Changji People’s Hospital, Changji 831100, China; xuejin202603@163.com; 3 Department of General Surgery, Xiangya Hospital, Central South University, 87 Xiangya Road, Kaifu District, Changsha 410017, China; fadaxia@csu.edu.cn (F.X.); ouyangxiwu@csu.edu.cn (X.O.); 4 Department of Oncology, The Second Affiliated Hospital of Xinjiang Medical University, Urumqi 830018, China

**Keywords:** papillary thyroid carcinoma, cell proliferation, lactate metabolism, immune infiltration, drug sensitivity

## Abstract

**Objectives**: The mechanism of action of genes related to lactate metabolism in papillary thyroid carcinoma (PTC) is still unclear. In this study, key genes that play a role in PTC were selected from the known genes related to lactate metabolism, and their roles in promoting lactate metabolism in PTC cells were investigated. **Methods**: Through bioinformatics analysis and cell experiments, the roles of the relevant genes in lactate metabolism and their roles in the occurrence and development of PTC were verified. **Results**: Through bioinformatics analysis, 12 candidate genes were obtained. Through qRT-PCR experiments, it was confirmed that the expressions of *TIMP1* and *DPP4* were higher in thyroid papillary carcinoma than in normal PTC cells. By inhibiting the expression of *TIMP1* and *DPP4* using siRNA, the invasion and proliferation abilities of PTC could be reduced. Compared with normal thyroid cells, the contents of lactic acid and LDHA in PTC cells were higher. Knocking down the expression of *TIMP1* and *DPP4* would reduce the lactate production ability of PTC cells, and *TIMP1* and *DPP4* promoted the accumulation of lactate in PTC cells.**Conclusions**: In this study, by screening the differentially expressed lactate metabolism genes in PTC, different prognostic subtypes were constructed based on the molecular expression patterns. Multi-group student’s t-tests were conducted on the differential signaling pathways and tumor immune regulation of the prognostic subtypes, and a PTC prognosis prediction model was constructed. It was further confirmed that the lactate metabolism genes *TIMP1* and *DPP4* are highly expressed in PTC and can regulate the proliferation, invasion, metastasis and lactate metabolism of PTC cells.

## Simple Summary

The genes related to lactate metabolism play a significant role in the occurrence and development of various tumors. The mechanism of their action in papillary thyroid carcinoma (PTC) still needs further research and exploration. In this study, key genes related to lactate metabolism that function in PTC were selected from the known genes related to lactate metabolism. Bioinformatics analysis, experimental verification, and exploration of the mechanism of these genes in PTC were conducted, and their role in promoting lactate metabolism in PTC cells was investigated. The results showed that compared with normal PTC cells, *TIMP1* and *DPP4* were highly expressed in thyroid papillary carcinoma. Silencing the expression of *TIMP1* and *DPP4* with siRNA could reduce the invasion and proliferation ability of PTC cells. Compared with normal thyroid cells, the content of lactate and LDHA in PTC cells was higher. Knocking down the expression of *TIMP1* and *DPP4* reduced the lactate production ability of PTC cells, and *TIMP1* and *DPP4* promoted the accumulation of lactate in PTC cells.

## 1. Introduction

Thyroid cancer is the most common endocrine malignancy worldwide [1], accounting for 3.4% of all diagnosed tumors [2]. Papillary thyroid carcinoma (PTC) is the most common histological subtype of thyroid cancer, accounting for 90% of new cases [3]. Surgery combined with thyroid-stimulating hormone (TSH)-suppression therapy or radioactive iodine therapy is the preferred treatment for PTC, and the prognosis of patients is usually good, but the rate of cervical lymph node metastasis is high, which may lead to local recurrence. Some patients may also experience distant metastasis and develop resistance to iodine-131, resulting in a poor prognosis [4]. Therefore, how to identify specific PTC patients and adopt specific treatment methods for different patient conditions has become an important research topic at present.

The most direct consequence of aerobic glycolysis is the increase in intracellular and extracellular lactate concentrations [5]. The occurrence of aerobic glycolysis is attributed to the increased demand for ATP metabolism in proliferating cells (such as tumor cells) [6], and the accumulation of lactate in the microenvironment is a characteristic of inflammatory diseases and cancer [7]. Under normal circumstances, lactate is mainly converted into pyruvate through aerobic respiration and enters the mitochondria for further metabolism. However, in tumor cells, due to their high metabolic requirements and micro-oxic environment, anaerobic glycolysis significantly increases, leading to excessive lactate production [8]. The accumulation of lactate not only alters the metabolic characteristics of tumor cells but also promotes the invasiveness and metastatic ability of tumor cells by establishing a low pH environment. Moreover, lactate has been proven to affect the function of immune cells in the tumor microenvironment, further promoting the development of cancer.

Lactate metabolism-related genes play a crucial role in various tumor types. For example, the expression of lactate dehydrogenase (LDH) and lactate transporter (LT) is closely related to the invasiveness and metastatic potential of tumors. However, the specific mechanism of these genes in papillary thyroid carcinoma remains unclear.

Lactate may be related to the invasion and prognosis of thyroid cancer. Lactate can stimulate the migration, invasion and tumor angiogenesis of thyroid cancer cells, and may promote the invasion and metastasis process of tumors by regulating the activity of transcription factors and signaling pathways [9]. Lactate can also promote the formation of the immune escape network, allowing tumor cell proliferation to be uncontrolled [10]. The accumulation of lactate can induce the differentiation of myeloid-derived suppressor cells (MDSC), regulatory T cells (Treg), and tumor-associated macrophages (TAM), stimulating their biological activity, and subsequently secreting immunosuppressive factors, inhibiting the immune response of natural killer cells (NK cells) and T cells, helping tumor cells evade immune surveillance and acquire unlimited growth potential [11]. Therefore, lactate metabolism plays an important role in the occurrence and development of thyroid cancer (TC) and may become a new therapeutic target. Regulating the tumor acidification environment or interfering with lactate metabolism-related signaling pathways may help in the treatment of thyroid cancer.

This study screened and verified differentially expressed lactate metabolism-related genes (DE-LRGs), and finally determined *TIMP1* and *DPP4* as two significantly differentially expressed lactate metabolism-related genes as experimental genes. The relationship between these genes and lactate metabolism was explored, and the influence of gene expression on their occurrence and development was verified. Regression analysis was used to screen genes related to the prognosis of papillary thyroid carcinoma. Lasso regression analysis was used to obtain the optimal risk score value for each sample for subsequent related analysis. Based on the median of the risk score, patients were divided into high-risk and low-risk groups, and the K-M curve was used for analysis. A prediction model was constructed based on these genes. This model demonstrated excellent predictive performance in both the training set and the internal validation set.

## 2. Materials and Methods

### 2.1. Cell Culture

In this study, human derived papillary thyroid carcinoma cell lines, including TPC, IHH4, BPCAP, and normal thyroid cell line NTHY, were used as cells. All cells are from Fenghui Biotechnology Company (Yuelu Subdistrict, Changsha City, Hunan Province, China). To ensure the optimal state of cells during cell culture and the reproducibility of experimental data, DMEM/1640 basic medium (90%), premium fetal bovine serum (5 mL), and penicillin streptomycin mixed antibiotics (P/S, 0.5 mL) were used as the culture medium.

Identification Declaration: “All human cell lines have been identified through STR typing, and the identification reports are valid for three years.” Pollution Control Declaration: “The cells used in the experiment have been tested and confirmed to be free of mycoplasma contamination.”

The cells were cultured in a constant temperature incubator at 37°C and 5% CO_2_. To ensure that the cells used in the experiment have stable biological characteristics, all experiments were conducted using cells that have been passaged for 3–8 generations.

### 2.2. Differential Expression LRGs Screening

The RNA-seq dataset was statistically analyzed using the limma package to identify the differentially expressed genes between the healthy and diseased samples. The criteria for selecting the differentially expressed genes were: adj.*p*.Val < 0.05 and |logFC| > 0.58. The *p*-values were adjusted for multiple testing using the Benjamini–Hochberg (BH) false discovery rate (FDR) method to control type I errors. Through the analysis of the expression profile of TCGA-papillary thyroid carcinoma, the differentially expressed genes related to PTC were screened out. The obtained results were intersected with the genes related to lactate metabolism to obtain the differentially expressed LRGs in PTC.

### 2.3. Obtaining Prognostic-Related Genes and Constructing Prediction Models

Select prognostic-related genes and use Lasso regression to further construct a prognostic-related model.

### 2.4. Exploration of Specific Signal Mechanisms in Prognostic Models

We use GSVA and GSEA enrichment analysis for exploration. GSVA enrichment analysis downloaded genes from the molecular signatures database, and GSVA performed gene set enrichment analysis (GSEA) on the expression profile of PTC patients; http://www.broadinstitute.org/gsea (accessed on 28 April 2025). To search for DE LRGs between high-risk and low-risk groups of patients.

### 2.5. Exploring the Clinical Predictive Value of Multi-Omics Models

The CIBERSORT algorithm was used to analyze RNA seq data from different subgroups of papillary thyroid carcinoma patients, in order to infer the relative proportions of 22 immune infiltrating cells. Pearson correlation analysis was performed on risk score values and immune cell content, and *p* < 0.05 was considered statistically significant.

We use the largest pharmacogenomics database (GDSC Cancer Drug Sensitivity Genomics Database, https://www.cancerrxgene.org/ accessed on 28 April 2025). We use the R software package (R4.5.0) “pRRophetic” to predict the chemotherapy sensitivity of each tumor sample and regression to obtain IC50 estimates for each specific chemotherapy drug treatment, and we conduct 10 cross validation tests on the GDSC training set to verify the regression and prediction accuracy. All parameters have been set to default values.

### 2.6. Screening of Candidate Experimental Genes

The lactate metabolism-related genes with differential expression were sorted based on the fold change value (logFC) and expression abundance, resulting in 12 candidate genes (logFC > 2, sorted by expression abundance from high to low). Combining the search results of these 12 genes in tumors and lactate metabolism, *TIMP1*, *SLPI*, *GDF15*, *TGFA*, *LCN2*, and *DPP4*, which have relatively high correlations, were selected as the experimental genes.

### 2.7. Quantitative Real-Time Polymerase Chain Reaction (qRT-PCR)

The total RNA was extracted using TRIzol reagent. Of the total RNA, 2 μg was used for reverse transcription. The experiment was conducted using the Bio-Rad CFX96 system, and the expression of mRNA was calculated. The relative expression was evaluated using the ΔΔCt value. All primers were purchased from Integrated DNA Technologies (Coralville, IA, USA).

### 2.8. Transwell

Add 100 microliters of cell suspension to the Transwell chamber and add 600 microliters of DMEM medium containing 20% serum to the 24-well plate. When inoculating the culture plate, be sure to avoid forming bubbles. Set up the control group (*TIMP1*), *TIMP1* knockdown group 1, *TIMP1* knockdown group 2, *DPP4* knockdown group 1, *DPP4* knockdown group 2, and control group (*DPP4*). Each group has 3 replicate samples. Use two papillary thyroid cancer cell lines (TPC, IHH4) for the experiment. After inoculation, culture the cells routinely for 24 h. Wash with PBS and stain with crystal violet solution for a few minutes. Use a cotton swab to wipe off the migrating cells on the upper layer, then wash with PBS three times. Observe the cells in each well under a fluorescence inverted microscope, count them, take the average value, and select the images with better migration effects for photography and statistical analysis.

### 2.9. CCK8

A certain number of cells were inoculated into a 96-well cell culture plate (2000 cells per well for the cell proliferation experiment). The plate was placed in an incubator for one day. Meanwhile, a blank group was set up (the blank group contained only the culture medium and CCK-8 Solution). The 96-well cell culture plate was placed in a cell incubator (37°C, 5% CO_2_) for incubation for an appropriate period of time. The samples of the first day of the detection group and the blank group were added to each well of the 96-well plate (20 μL of CCK-8 Solution per well) and incubated at 37 °C for 1 h. After incubation, the absorbance values were measured using an enzyme-labeled instrument. The above operation was repeated at 24 h, 48 h, and 72 h. After 24 h, the cells in the 96-well plate were changed to another one. The absorbance at 450 nm of cells in each of the six groups (NC group, *TIMP1* knockdown group 1, *TIMP1* knockdown group 2, NC group, *DPP4* knockdown group 1, *DPP4* knockdown group 2) on days 1, 2, 3, and 4 was recorded. When the differences between the duplicate wells were too large, they were excluded. The average values of the duplicate wells were taken and recorded for plotting.

### 2.10. Lactic Acid Test

Set up the blank group, the standard group and the sample group. In the blank group, add 5 microliters of deionized water and 200 microliters of the detection buffer, mix well, incubate at 37 °C for 5 min, and then add 40 microliters of the enzyme solution; in the standard group, add 5 microliters of the lactic acid standard solution and 200 microliters of the detection buffer, mix well, incubate at 37 °C for 5 min, and then add 40 microliters of the enzyme solution; in the sample group, add 5 microliters of the sample and 200 microliters of the detection buffer, mix well, incubate at 37 °C for 5 min, and then add 40 microliters of the enzyme solution. After all groups are mixed, measure the absorbance at 546 nanometers using a spectrophotometer and calculate the lactic acid content.

### 2.11. Lactate Dehydrogenase Test

According to the experimental requirements, different groups were set up. The cells were cultured for one day, washed with PBS, 120 μL of cell lysis solution was added, incubated in the incubator for a few minutes, centrifuged after the incubation, and then 80 μL of the solution was added to the pre-prepared 96-well plate. Prepare an appropriate amount of LDH detection working solution according to the number of samples to be tested. Conduct experiments on approximately 50 samples, adding 1 mL of reaction substrate, 100 μL of INT solution, 150 μL of enzyme solution and 2.75 mL of reaction buffer (total volume 4 mL). Add the LDH detection working solution to the sample wells, 80 μL per well, and gently shake to mix. Place the cells in a light-protected incubator for a few minutes, then detect the absorbance of LDH.

### 2.12. Statistical Analysis

All experimental data are presented as mean ± standard deviation (SD) from at least three independent biological replicates. Statistical comparisons between two groups were performed using a two-tailed Student’s t-test. Comparisons among multiple groups were conducted using one-way analysis of variance (ANOVA) followed by an LSD post hoc test. Kaplan–Meier curves were generated for survival analysis, and differences were compared using the log-rank test. Univariate and multivariate Cox regression analyses were applied to identify prognostic factors. Pearson correlation coefficient was used to evaluate linear correlations. All statistical tests were two-sided, and *p* < 0.05 was considered statistically significant. Data processing and statistical analyses were performed using R software (version 4.0) and GraphPad Prism (version 10.0.2).

## 3. Results

### 3.1. Analysis of mRNA Expression Levels (Patients vs. Controls)

**Figure 1:**
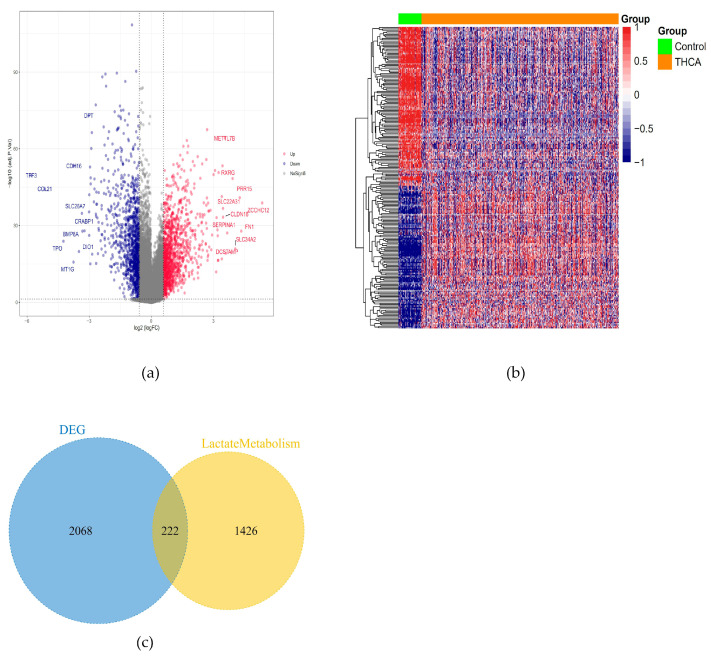
The mRNA expression level and genes related to lactate metabolism: (**a**) Volcano plot showing 2290 differentially expressed genes between PTC and normal tissues, including 1213 upregulated and 1077 downregulated genes (adj.*p*.Val < 0.05, |logFC| > 0.58). (**b**) Heatmap displaying the top 50 dysregulated genes in PTC and normal samples. (**c**) Venn diagram identifying 222 DE-LRGs as the intersection of PTC-related DEGs and known lactate metabolism-related genes. These DE-LRGs were used for subsequent clustering, prognostic modeling, and candidate gene screening.

The limma software (R4.5.0) package was used to conduct statistical analysis on the RNA sequencing data set, aiming to identify differentially expressed genes between diseased and healthy samples. The selection criteria were an adjusted *p*-value < 0.05 and a log expression fold change > 0.58. After analyzing the expression profile of TCGA papillary thyroid carcinoma, a total of 2290 related differentially expressed genes were obtained. (Figure 1a,b). Among them, there were 222 genes related to lactate metabolism (Figure 1c).

### 3.2. DE-LRGs Undergo Consistent Cluster Analysis

**Figure 2:**
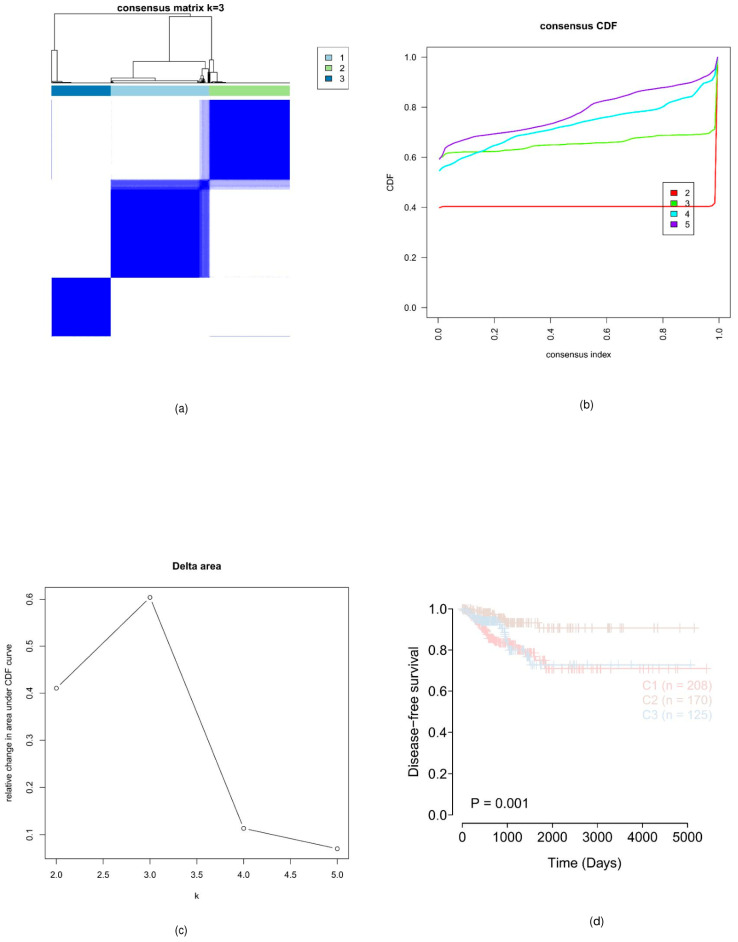
The results of the consistency clustering analysis show that, compared with C1 and C3, C2 has a higher survival probability; *p* < 0.001. (**a**,**b**) Consensus matrix and cumulative distribution function (CDF) curves indicating the optimal clustering number k = 3. (**c**,**d**) Kaplan–Meier disease-free survival (DFS) curves showing that the C2 subtype exhibited significantly better prognosis than C1 and C3 subtypes (*p* < 0.001). These results suggest that lactate metabolism-based clustering can reflect the prognostic heterogeneity of PTC.

The expression consistency of 222 DE-LRGs in the PTC cohort was analyzed using the R software package ConsensusClusterPlus. The results showed that the consistency was the highest when the clustering number k was 3 (Figure 2a,b). The survival and prognosis differences in the three subtypes were evaluated using the K-M curve. The results indicated that the survival probability of the C2 subtype was significantly higher than that of the C1 and C3 subtypes (Figure 2c,d), suggesting that the combination of the expression of genes related to tissue lactate metabolism can effectively predict individuals with a high risk of recurrence.

### 3.3. Evaluation of Subtype Molecular Characteristics

**Figure 3:**
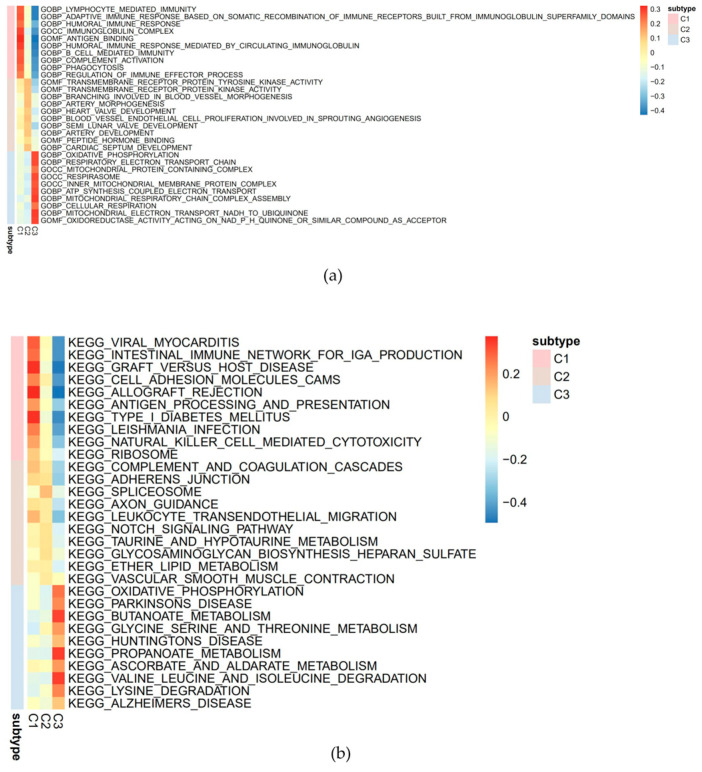
Evaluation results of subtype molecular characteristics: (**a**) GO enrichment analysis showing the top activated pathways in the C2 subtype, mainly involving transmembrane receptor protein tyrosine kinase activity. (**b**) KEGG enrichment analysis revealing that the C2 subtype was enriched in the Notch signaling pathway and other cancer-related pathways. These pathways may underlie the favorable prognosis of the C2 subtype.

This study quantitatively analyzed GO and KEGG pathways using the ssGSEA algorithm and found significant differences in multiple pathways among different subgroups. The GO analysis revealed that the high-scoring pathways for the C2 subtype included transmembrane receptor protein tyrosine kinase activity and other pathways (Figure 3a); the KEGG analysis indicated that the high-scoring pathways for the C2 subtype encompassed the Notch signaling pathway and other pathways (Figure 3b).

### 3.4. Obtain Prognostic-Related Genes and Construct a Prediction Model

**Figure 4:**
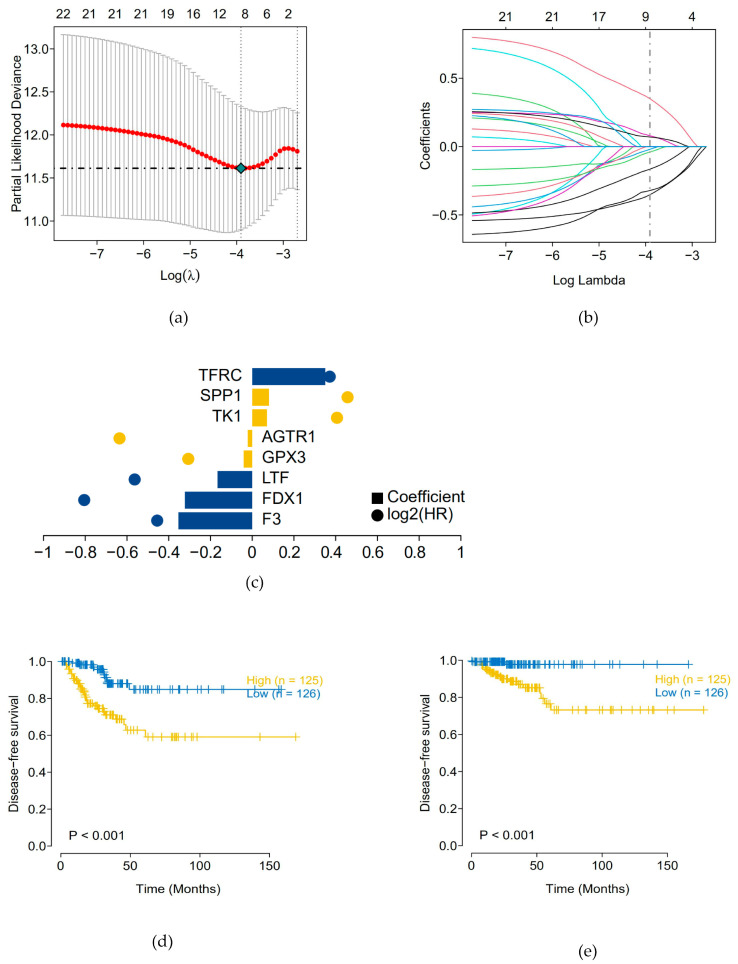
The screening results of characteristic genes in papillary thyroid carcinoma and the DFS of the training set and test set: (**a**–**c**) Lasso regression analysis identifying 8 key prognostic genes from DE-LRGs. (**d**,**e**) Kaplan–Meier DFS curves demonstrating that the high-risk group had significantly shorter survival than the low-risk group in both training and validation sets (*p* < 0.001). Data are presented as the mean ± SD; comparisons were performed using the log-rank test.

**Figure 5:**
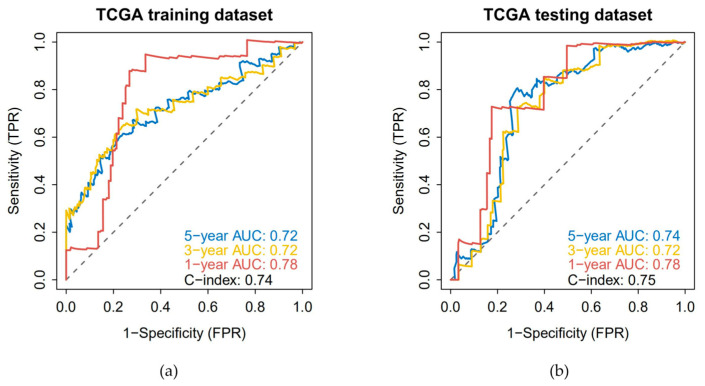
Model evaluation results: (**a**) The ROC curve results show that the AUC values calculated for the three periods (1 year, 3 years, and 5 years) in the training set are greater than 0.7, indicating that the model has good validation efficacy. (**b**) The results of the ROC curve showed that the AUC values calculated for the 1-year, 3-year, and 5-year periods in the test set were greater than 0.7, indicating that the model has good validation performance.

Clinical information of patients with papillary thyroid carcinoma was collected. Through Cox univariate regression using the DE-LRGs set, 22 prognostic-related genes were selected. Then, the characteristic genes were determined by Lasso regression (Figure 4a–c). The TCGA patients were randomly divided 1:1 into the training set and the internal validation set. The sample risk score was obtained through Lasso regression (risk score = F3 × (−0.3524) + FDX1 × (−0.3216) + LTF × (−0.1652) + GPX3 × (−0.0400) + AGTR1 × (−0.0201) + TK1 × 0.0700 + SPP1 × 0.0798 + TFRC × 0.3502). The patients were divided into high-risk and low-risk groups based on the median score. Kaplan–Meier analysis showed that the disease-free survival time of the high-risk group in both the training set and the validation set was significantly lower than that of the low-risk group (Figure 4d,e); the ROC curve confirmed that the AUC values of model 1, 3, and 5 years were all ≥0.7 (Figure 5a,b), indicating good predictive efficacy.

### 3.5. Constructing the Nomogram and the Calibration Curve

**Figure 6:**
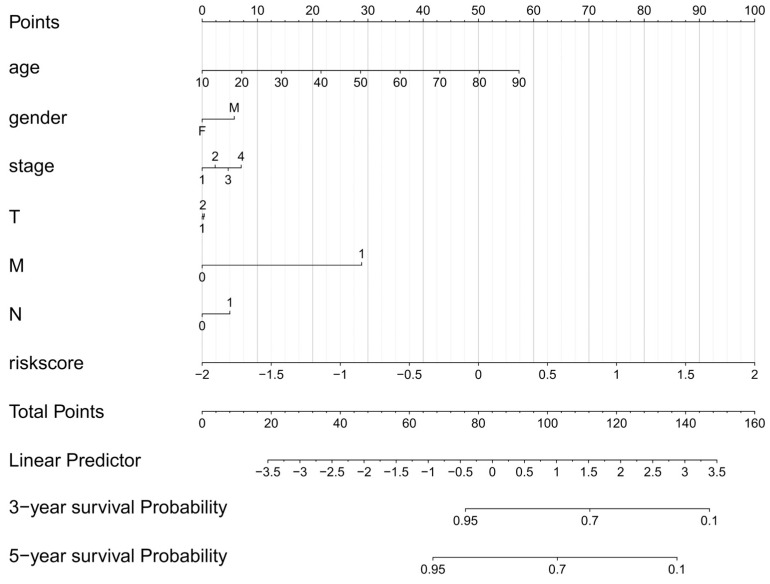
Nomogram integrating risk score, age, and M stage to generate a quantitative scoring system for individualized prognosis prediction. Each variable was assigned a point value, and the total points corresponded to the predicted survival probability.

**Figure 7:**
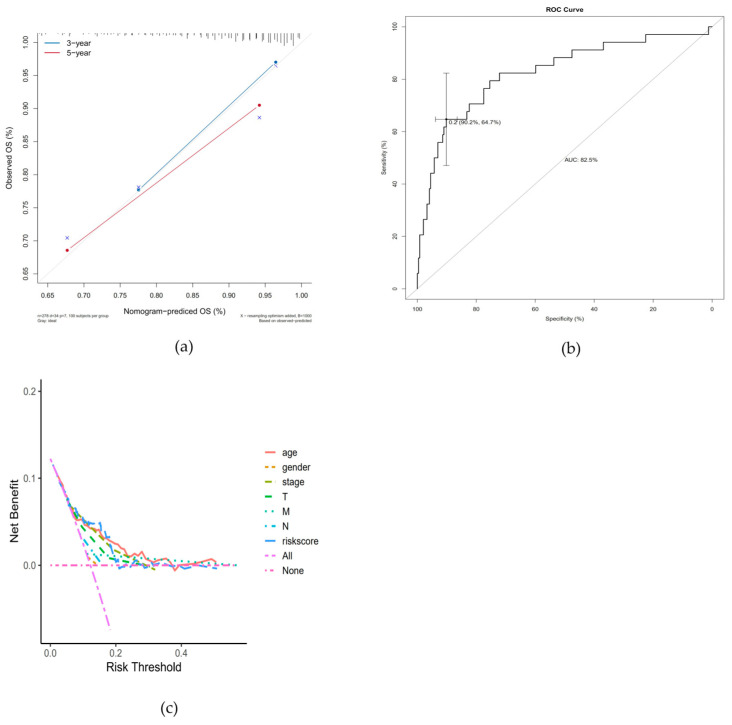
Calibration plots for 3-year and 5-year DFS showing excellent consistency between the nomogram-predicted survival and actual observed survival, indicating high accuracy of the predictive model. (**a**) Calibration curves of the nomogram for 3-year and 5-year OS. The diagonal dashed line represents the ideal prediction, and the solid lines represent the actual predictive performance of the nomogram. (**b**) ROC curve of the nomogram for OS prediction, with an AUC of 82.5%. (**c**) Decision curve analysis (DCA) of the nomogram and other clinical factors. The y-axis measures the net benefit, and the x-axis shows the risk threshold. The riskscore curve demonstrates superior net benefit compared with other clinical factors, the "treat-all" strategy, and the "treat-none" strategy across a wide range of threshold probabilities.

The clinical information and risk scores of patients in the high-risk and low-risk groups were integrated. The results of the regression analysis were presented in the form of a nomogram. The results of the logistic regression analysis indicated that in all of our samples, the clinical indicator values of age, M, and risk score contributed to multiple scoring processes. The risks core value and the distribution of the clinical indicator age obtained through model analysis had a higher contribution in the scoring process of the prediction analysis (Figure 6). The prediction analysis for the three-year and five-year periods of thyroid papillary carcinoma revealed that the predicted DFS was in good agreement with the observed DFS (Figure 7a–c). This indicates that the model has certain predictive value. 

### 3.6. The Results of the Signaling Pathways Involved in the Prognosis Model

**Figure 8:**
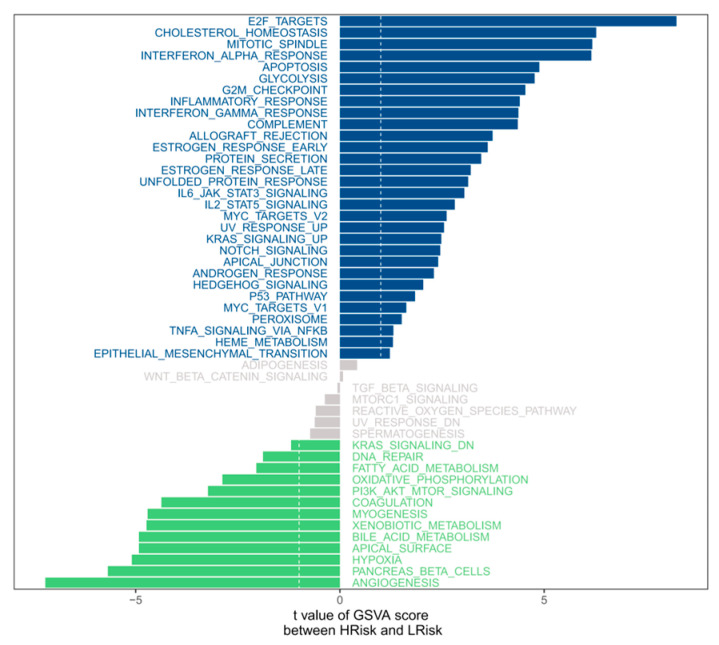
GSVA showing that the high-risk group was mainly enriched in E2F targets, cholesterol homeostasis, and mitotic spindle pathways, which are closely associated with cell proliferation and malignant progression.

**Figure 9:**
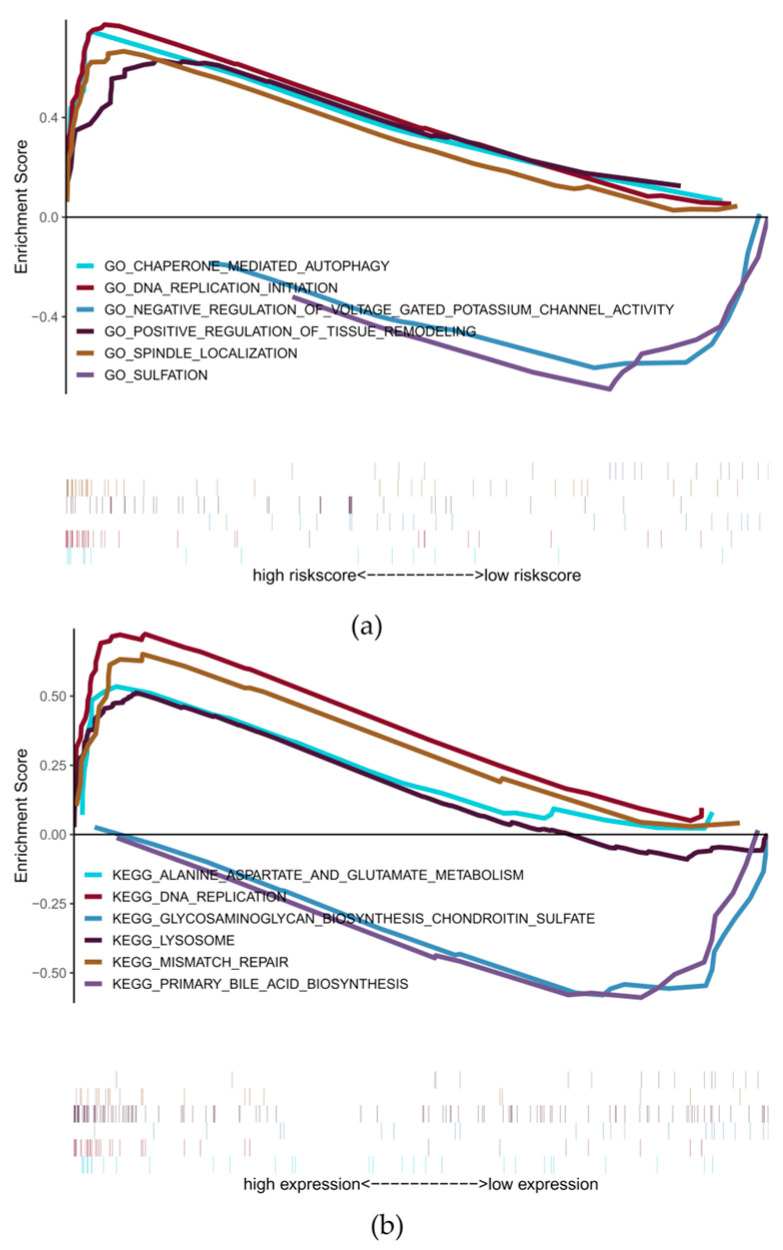
The results of GSEA GO analysis and GSEA KEGG analysis results: (**a**) GO terms enriched in the high-risk group, including chaperone-mediated autophagy and DNA replication initiation. (**b**) KEGG pathways including alanine, aspartate and glutamate metabolism, and DNA replication. These pathways may mediate the prognostic effect of lactate metabolism genes.

The study explored the signaling pathways involved in different risk group models and initially identified the potential mechanisms by which the risk score affects the development of PTC. The GSVA analysis mainly enriched e2f targets, cholesterol homeostasis, mitotic spindle, etc. (Figure 8). Through GSEA, it was found that many related pathways were significantly enriched. The GO-enriched pathways included chaperone-mediated autophagy, DNA replication initiation, etc.; the KEGG-enriched pathways included alanine aspartate and glutamate metabolism, DNA replication, etc. (Figure 9a,b). For some of the highly significant pathways, they were presented in a concentrated manner. The results showed that the disturbances of these signaling pathways in patients with high and low risk groups might be associated with the prognosis of PTC patients.

### 3.7. Exploring the Clinical Predictive Value of the Model

The association between the risk score and tumor immune infiltration was analyzed, and the potential molecular mechanisms underlying the influence of this on the progression of papillary thyroid carcinoma were further explored. The results showed that there was heterogeneity in the infiltration levels of different immune factors (Figure S1), and there were multiple significant correlations among the immune factors (Figure S2). The levels of immune components such as activated NK cells were significantly higher in the low-risk group than in the high-risk group, while immature B cells and Tregs were significantly lower (Figure S3); the risk score was significantly positively correlated with Tregs and M0-type macrophages, and significantly negatively correlated with CD8+ T cells and activated NK cells (Figure S4). Based on the GDSC database, using the pRRophetic package to predict chemotherapy sensitivity, it was found that the risk score was significantly correlated with the sensitivity to drugs such as axitinib (Figure S5). At the same time, the distribution of mutation types in the high and low expression groups of the patient samples was not completely consistent (Figure S6). At the same time, the level of tumor mutational burden (TMB) also had significant differences between the groups (Figure S7). Through the analysis of tumor immune dysfunction and rejection, it was found that there were differences in the high-risk and low-risk groups, and Responder, Exclusion, etc., showed significant differences between the high-risk and low-risk groups (Figure S8a,b). The above results indicate that lactate metabolism may regulate the malignancy of tissues through complex immune regulatory mechanisms, thereby influencing the recurrence risk of patients (Figures S1–S8 can be found in the Supplementary Document).

### 3.8. Selection of Alternative LRGs

**Figure 10:**
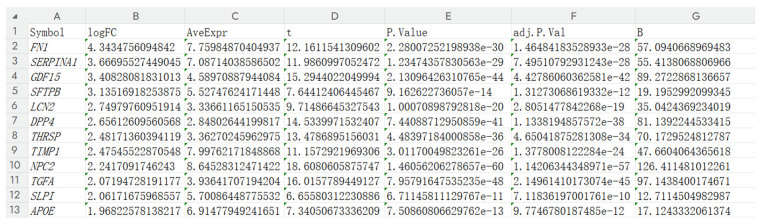
After conducting a detailed search on 222 DE-LRGs, it was found that there were 6 genes that have been extensively studied in other tumors and are related to lactate metabolism but have not been investigated in thyroid papillary carcinoma. These genes are *TIMP1*, *DPP4*, *SLPI*, *GDF15*, *TGFA*, and *LCN2.*

The above studies have confirmed that genes related to lactate metabolism play an important role in PTC, participating in a complex regulatory network. Different gene expression patterns can be used to screen patients with a higher risk of recurrence. Based on bioinformatics research, in order to further verify the role of lactate metabolism genes in PTC, we screened the corresponding genes for functional validation studies. The expression information of 222 LRGs was sorted according to LogFC, and the genes with more obvious differential expression were selected as candidate genes for further screening (Figure 10). The gene + lactate metabolism + cancer was searched one by one on PubMed, and it was found that in other tumors, studies related to lactate metabolism were more numerous and not involved in thyroid papillary carcinoma. There were six genes that were not studied in thyroid papillary carcinoma but were related to lactate metabolism in other tumors, namely *TIMP1*, *DPP4*, *SLPI*, *GDF15*, *TGFA*, and *LCN2.* These genes were selected as pre-selected genes for qRT-PCR verification.

### 3.9. The Results of qRT-PCR Verification

**Figure 11:**
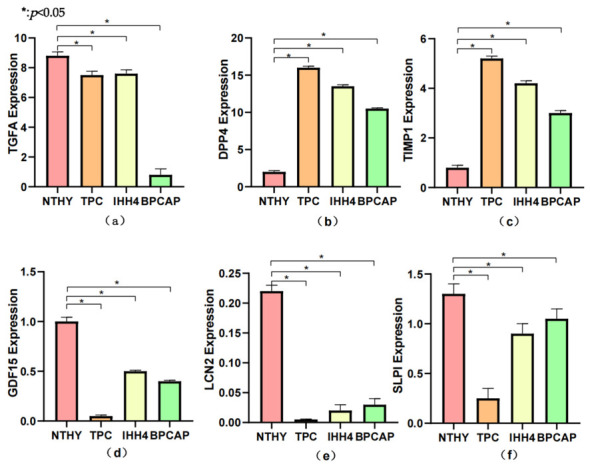
The expression of genes in thyroid cell lines and papillary thyroid carcinoma cell lines; *p* < 0.05: The qRT-PCR results showed that compared with the expression in normal thyroid cell lines, *TIMP1* and *DPP4* were upregulated in the thyroid papillary carcinoma cell lines *TPC*, *IHH4*, and *BPCAP*; *GDF15*, *LCN2*, *SLP1*, and *TGFA* were also upregulated in the thyroid papillary carcinoma cell lines. *TGFA* was highest in NTHY (8.8 ± 0.3) and significantly lower in TPC (7.5 ± 0.3), IHH4 (7.6 ± 0.3), and BPCAP (0.8 ± 0.2) cells. *DPP4* was markedly upregulated in PTC lines relative to NTHY (1.8 ± 0.2), peaking in TPC (16.0 ± 0.3), followed by IHH4 (13.5 ± 0.3) and BPCAP (10.5 ± 0.2). *TIMP1* showed a similar trend to *DPP4*, with elevated levels in TPC (5.2 ± 0.2), IHH4 (4.2 ± 0.1), and BPCAP (3.0 ± 0.1) compared to NTHY (0.8 ± 0.1). *GDF15* was downregulated in PTC cells: NTHY (1.0 ± 0.05) > IHH4 (0.5 ± 0.03) > BPCAP (0.4 ± 0.02) > TPC (0.05 ± 0.02). *LCN2* expression was largely restricted to NTHY (0.22 ± 0.01), with negligible levels in all PTC lines (≤0.03). *SLPI* was significantly reduced only in TPC (0.3 ± 0.1) versus NTHY (1.3 ± 0.1); IHH4 (0.9 ± 0.1) and BPCAP (1.05 ± 0.1) showed intermediate expression.(**a**) The expression of *TGFA* in four cell lines. (**b**) The expression of *DPP4* in four cell lines. (**c**) The expression of *TIMP1* in four cell lines. (**d**) The expression of *GDF15* in four cell lines. (**e**) The expression of *LCN2* in four cell lines. (**f**) The expression of *SLPI* in four cell lines.

The repeated qRT-PCR experiment verification results showed that compared with normal thyroid cells, *TIMP1* and *DPP4* were upregulated in the thyroid papillary carcinoma cell lines TPC, IHH4, and BPCAP, which was consistent with the results of bioinformatics screening; *SLPI*, *GDF15*, *TGFA*, and *LCN2* were downregulated in the thyroid papillary carcinoma cell lines, which was inconsistent with the results of bioinformatics analysis, so they were excluded (Figure 11).

### 3.10. The Effects of TIMP1 and DPP4 on Lactate Metabolism

**Figure 12:**
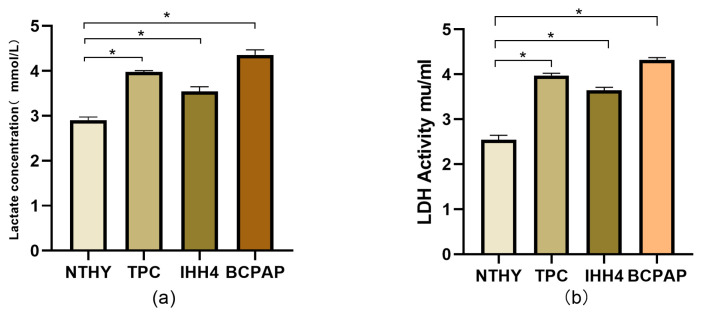
(**a**) Lactic acid content of NTHY, BPCAP, TPC, IHH4 cell lines; compared with the NTHY group, the expression levels in TPC (3.98 ± 0.06), IHH4 (3.53 ± 0.09), and BPCAP (4.35 ± 0.10) groups were significantly increased (* *p* < 0.05 for TPC, IHH4 and BPCAP vs. NTHY). Compared with the normal thyroid cell line NTHY, the thyroid papillary carcinoma cell lines (BPCAP, TPC, IHH4) have a stronger lactate metabolism and excessive lactate accumulation. These results indicate that *TIMP1* and *DPP4* are critical promoters of lactate metabolism in PTC. Given the reported role of *TIMP1* in regulating glycolysis via the CD63 pathway, the reduced lactate production upon *TIMP1* knockdown further implies suppressed glycolytic activity in PTC cells. (**b**) LDH activity of NTHY, TPC, IHH4 and BPCPA cell lines; compared with the NTHY group (2.53 ± 0.09), the expression levels in the TPC group (3.97 ± 0.07), the IHH4 group (3.62 ± 0.08), and the BPCAP group (4.32± 0.06) were significantly decreased (compared with the control group NTHY, the expression levels in the TPC, IHH4, and BPCAP groups were significantly upregulated (* *p* < 0.05)). Compared with normal thyroid cell lines, the lactate dehydrogenase content in thyroid papillary carcinoma cell lines is higher.

Four thyroid cell lines were cultured. After 24 h, the cell culture supernatant was centrifuged at 2500 rpm for 10 min. The sample was loaded onto a 96-well plate using the Servicebio lactic acid detection kit. The OD value at 546 nm was measured by spectrophotometry to calculate the lactate content. The results showed that compared with the normal thyroid cell line NTHY, the thyroid papillary carcinoma cell lines (BPCAP, TPC, IHH4) had stronger lactate metabolism and excessive accumulation of lactate (Figure 12a).

The Servicebio Lactate Dehydrogenase Cytotoxicity Detection Kit can be used for cell LDH detection. Measuring the LDH content can indirectly reflect the strength of lactate metabolism. The test results show that compared with normal thyroid cell lines, the LDH content in thyroid papillary carcinoma cell lines is higher. (Figure 12b)

**Figure 13:**
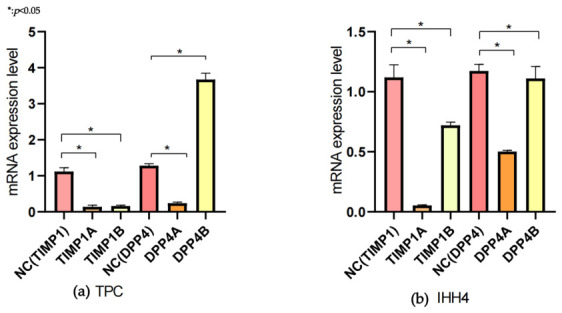
The expression of the gene after transfection in TPC and IHH4 cells: (**a**) The results showed that in the thyroid papillary carcinoma cell line TPC, compared with the *TIMP1*B group and the *DPP4*B group, the *TIMP1*A group and the *DPP4*B group performed better. (**b**) The results showed that in the thyroid papillary carcinoma cell line IHH4, compared with the *TIMP1*B group and the *DPP4*B group, the *TIMP1*A group and the *DPP4*B group performed better. In TPC cells, compared with the corresponding negative control (NC) groups, the mRNA expression of TIMP1 was significantly downregulated in TIMP1A and TIMP1B knockdown groups (1.12 ± 0.08 vs. 0.13 ± 0.03, 0.12 ± 0.02, *p* < 0.05), while the mRNA expression of DPP4 was significantly decreased in DPP4A knockdown group (1.28 ± 0.07 vs. 0.21 ± 0.04, *p* < 0.05) and remarkably upregulated in DPP4B overexpression group (1.28 ± 0.07 vs. 3.68 ± 0.12, *p* < 0.05). In IHH4 cells, the mRNA expression of TIMP1 was significantly reduced in the TIMP1A knockdown group (1.10 ± 0.11 vs. 0.05 ± 0.02, *p* < 0.05), while the TIMP1B group showed a moderate downregulation (1.10 ± 0.11 vs. 0.72 ± 0.04, *p* < 0.05). For DPP4, the mRNA level was significantly decreased in the DPP4A knockdown group (1.15 ± 0.06 vs 0.50 ± 0.03, *p* < 0.05) and notably increased in the DPP4B overexpression group (1.15 ± 0.06 vs. 1.09 ± 0.09, *p* < 0.05). All data are presented as the mean ± standard deviation (SD) of three independent experiments.

Compared with the control group, when the expressions of *TIMP1* and *DPP4* were downregulated, the lactate metabolism significantly decreased and the ability to accumulate lactate weakened (Figure 13a,b).

### 3.11. The Effects of TIMP1 and DPP4 on the Proliferation and Metastasis of PTC

**Figure 14:**
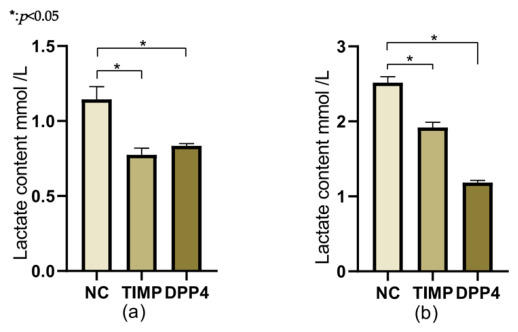
Changes in lactate content after downregulation of *TIMP1* and *DPP4*: (**a**) After the downregulation of *TIMP1* and *DPP4* in the TPC cell line, the lactate content decreased. (**b**) After the downregulation of *TIMP1* and *DPP4* in the IHH4 cell line, the lactate content decreased.

To further explore the impact of genes on cell proliferation and migration through regulating lactate metabolism, we conducted transfection to downregulate *TIMP1* and *DPP4* and then verified it using qRT-PCR. The results showed that in the thyroid papillary carcinoma cell lines TPC and IHH4, compared with the *TIMP1*B group and *DPP4*B group, the *TIMP1*A group and *DPP4*B group performed better for subsequent experiments. (Figure 14)

**Figure 15:**
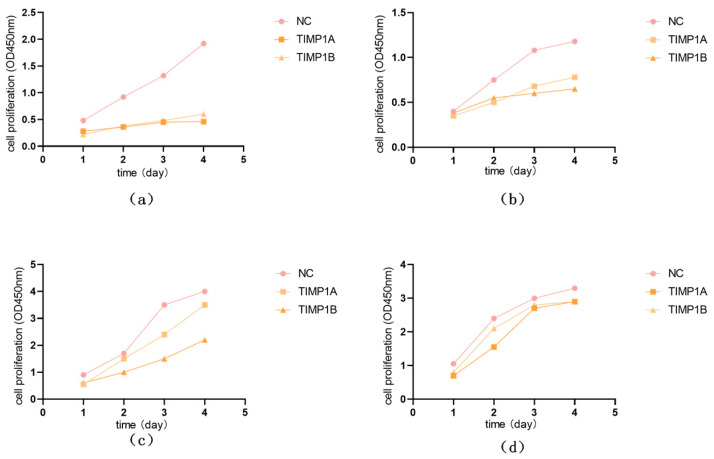
Downregulation of *TIMP1* and *DPP4* leads to changes in cell proliferation: (**a**) The changes in cell proliferation over time after the downregulation of *TIMP1* expression in the TPC cell line. (**b**) The changes in cell proliferation over time after the downregulation of *DPP4* expression in the TPC cell line. (**c**) The changes in cell proliferation over time after the downregulation of *TIMP1* expression in the IHH4 cell line. (**d**) The changes in cell proliferation over time after the downregulation of *DPP4* expression in the IHH4 cell line.

**Figure 16:**
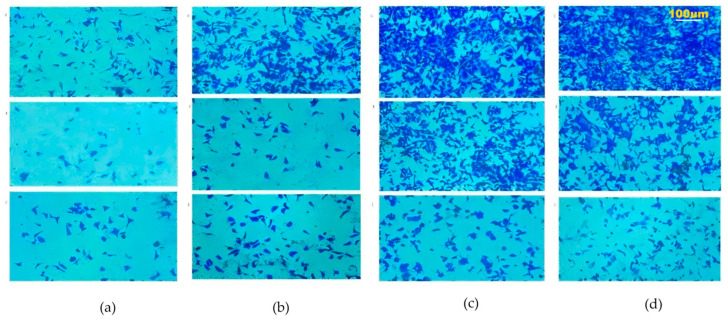
The changes in cell migration ability after downregulating *TIMP1* and *DPP4*: (**a**) After downregulating the expression of *TIMP1* in the TPC cell line using different si-RNA, the cell migration ability was significantly weakened. (**b**) After downregulating the expression of *TIMP1* in the IHH4 cell line using different si-RNA, the cell migration ability was significantly weakened. (**c**) After downregulating the expression of *DPP4* in the TPC cell line using different si-RNA, the cell migration ability was significantly weakened. (**d**) After downregulating the expression of *DPP4* in the IHH4 cell line using different si-RNA, the cell migration ability was significantly weakened. (Representative fields of thyroid cancer cells subjected to Transwell assays. Cells were fixed and stained with 0.1% crystal violet, then visualized under an optical microscope at 100× magnification (10× objective, 10× eyepiece). Scale bar = 100 μm.)

Compared with the control group (NC), when the expressions of *TIMP1* and *DPP4* were downregulated, the proliferation ability of TPC and IHH4 cell lines was weakened (Figure 15); when the expressions of *TIMP1* and *DPP4* were downregulated, the migration ability of TPC and IHH4 cell lines was also weakened (Figure 16).

## 4. Discussion

Existing studies have confirmed that genes related to lactic acid metabolism play a significant role in tumor prognosis [12,13,14,15,16]. For instance, in lung adenocarcinoma, it has been demonstrated that the lactic acid metabolism-related gene KRT81 plays a crucial role in the occurrence and development of lung adenocarcinoma. By establishing a nude mouse xenograft model and injecting A549 cells transfected with siNC and siRNA2, the tumor changes were closely monitored. The results confirmed that KRT81 plays an important role in the progression of lung adenocarcinoma [17]. Although extensive bioinformatics analysis and basic experimental research on lactic acid metabolism-related genes have been conducted in many tumors, studies on papillary thyroid carcinoma (PTC) are relatively scarce. The impact of lactic acid metabolism-related genes on lactic acid metabolism in PTC and their role in the proliferation and metastasis of PTC still require further investigation. 

The immune system plays a crucial role in preventing cancer and in the occurrence and development of cancer. Recent studies on immune infiltration in PTC have made many significant advances, especially in the research of major immune cells such as B cells and NK cells [18,19,20,21,22,23]. It has been found that these tumor-promoting immune cells are massively recruited in PTC and exhibit a tumor-promoting immune microenvironment phenotype [24,25,26]. This indicates that PTC can manipulate some immune cells, such as M2 macrophages, regulatory T cells, monocytes, neutrophils, dendritic cells, mast cells, and M0 macrophages, to suppress the body’s immune response to itself, which can also be called “damage”, ultimately enabling the tumor to evade immune surveillance and immune system attacks [27]. The analysis of the correlation between risk scores and tumor immune infiltration revealed that the distribution of immune factors varies among different samples, and there is a widespread and significant correlation among these factors.

In tumor-related research, the construction of predictive models has become an important task. The construction of predictive models has far-reaching and multi-level impacts, capable of integrating medical data from databases and serving as a tool for guiding clinical decision-making and scientific research. By constructing a predictive model based on disease-free survival, the aim is to predict the sensitivity of PTC patients to different drugs, tumor mutation burden, and immune treatment response, and to make preliminary predictions of the 1-year, 3-year, and 5-year disease-free survival of patients.

The lactate metabolism-related prognostic signature was established based on differentially expressed lactate metabolism-associated genes in PTC patients. *TIMP1* and *DPP4* were identified and selected as core candidate genes from the gene pool included in this prognostic model, representing key molecules linked to lactate metabolism and prognosis. Subsequent cellular experiments confirmed that *TIMP1* and *DPP4* were highly expressed in papillary thyroid carcinoma cells, and their knockdown suppressed cell proliferation, migration, and lactate production. These functional results experimentally verified the biological significance of the metabolic genes highlighted by the prognostic model. In turn, the clinical prognostic value of the model was supported by the in vitro phenotypes. Therefore, the bioinformatic analysis and experimental validation are highly complementary and integrated: the former provides predictive targets and clinical implications, while the latter confirms the functional mechanism of key genes in the model.

Notably, a previous study demonstrated that *TIMP1* regulates tumor glycolysis and extracellular acidification via the *TIMP1*-CD63 axis in breast cancer [28]. Consistent with this mechanism, our results showed that *TIMP1* knockdown significantly reduced lactate production and LDH activity in PTC cells, supporting *TIMP1* as a positive regulator of glycolytic metabolism. Similarly, *DPP4* has been implicated in metabolic reprogramming in multiple cancers [29,30], and our data further revealed that *DPP4* silencing also suppressed lactate accumulation in PTC cells. Although direct glycolytic flux measurements (e.g., ECAR) were not performed in this study, our lactate and LDH data are functionally consistent with attenuated glycolysis following *TIMP1* or *DPP4* depletion. We therefore infer that *TIMP1* and *DPP4* may synergistically promote glycolysis and lactate accumulation in PTC, with *TIMP1* acting potentially through the CD63 signaling axis; these mechanisms warrant further validation in future studies.

This study has demonstrated that genes related to lactate metabolism can serve as markers for predicting the disease-free survival period of PTC. The levels of immune cells, tumor microenvironment, and tumor burden may be involved in the pathogenesis of PTC, and it has been confirmed that genes such as *TIMP1* and *DPP4* related to lactate metabolism can regulate the lactate metabolism and proliferation and metastasis of PTC. However, this study still has some shortcomings. Firstly, due to the lack of external datasets for thyroid papillary carcinoma in this study, the prognostic model was not re-verified using external datasets. Secondly, the specific signaling pathways involved in the effects of *TIMP1* and *DPP4* on the occurrence and development of PTC and their impact on lactate metabolism need to be further explored. Further downstream experiments and animal experiments are needed to conduct further research. In future studies, we will improve these issues.

## 5. Conclusions

This study conducted DE-LRGs screening and validation to ultimately determine *TIMP1* and *DPP4* as the two lactate metabolism-related genes with relatively significant differential expression as the experimental genes. It explored the relationship between genes and lactate metabolism and verified the influence of gene expression and its impact on the occurrence and development of papillary thyroid carcinoma. The results showed that *TIMP1* and *DPP4* were highly expressed in PTC, and lactate metabolism was inhibited after downregulating the expression of *TIMP1* and *DPP4* genes, and the proliferation and migration ability of PTC were weakened.

Through clustering analysis of 222 DE-LRGs, it was found that the consistency was the highest when k = 3. The survival differences between subtypes were evaluated using the K-M curve, and the prognostic differences in different molecular subtypes were explored. The results showed that compared with C1 and C3, C2 had a better survival possibility.

Through single-factor Cox regression and Lasso regression analysis, differentially expressed genes related to lactate metabolism were screened, and a risk scoring model was constructed. This model demonstrated good predictive efficacy in both the training set and the internal validation set and could effectively predict the recurrence and metastasis risks of patients with papillary thyroid carcinoma (PTC). A nomogram was constructed by combining clinical indicators to provide an intuitive and accurate reference for clinical decision-making.

## Data Availability

This analysis compared the differentially expressed genes of the diseased condition from the TCGA database (https://portal.gdc.cancer.gov/, accessed on 28 April 2025), and the lactate metabolism-related gene set (Lactate Metabolism) was derived from the GeneCard database (https://www.genecards.org, accessed on 28 April 2025).The data for drug sensitivity analysis were obtained from the Pharmacogenomics Database (GDSC Cancer Drug Sensitivity Genomics Database, https://www.cancerrxgene.org/, accessed on 28 April 2025).

## Supplementary Materials

The following supporting information can be downloaded at: https://www.mdpi.com/article/10.3390/cancers18081264/s1, Figure S1: The distribution of immune levels of different immune factors in samples. Figure S2: The interrelationship among immune factors on; * *p* < 0.05, ** *p* < 0.01, *** *p* < 0.001. Figure S3: Immune factor levels in high-risk and low-risk groups; * *p* < 0.05, ** *p* < 0.01, *** *p* < 0.001. Figure S4: The relationship between risk score and immune cells; *p* < 0.001. Figure S5: The level of risk assessment is significantly correlated with the patients’ sensitivity to drugs such as Axitinib, Bicalutamide, Bortezomib, Bosutinib, Cisplatin and Cyclopamine. Figure S6: Distribution of mutation types in high-risk and low-risk groups results. Figure S7: The results showing the difference in tumor mutational burden between the high-risk group and the low-risk group indicated that the tumor mutational burden in the high-risk group was lower than that in the low-risk group. Figure S8: Differences in tumor immune function between high-risk and low-risk groups:There were significant differences in variables such as Responder and Exclusion between the high-risk and low-risk groups.

## Abbreviations

The following abbreviations are used in this manuscript:

DE-LRG	Differential expression lacate metabolism-related gene
DFS	Disease free survival
GEO	Gene Expression Omnibus
GO	Gene ontology
GSVA	Gene Set Variation Analysis
GSEA	Gene Set Enrichment Analysis
KEGG	Kyoto Genome Encyclopedia
LDH	lactate dehydrogenase
LMRGS	Lactate metabolism-related genes
LT	lactate transporter
MDSC	Myeloid-derived suppressor cells
PTC	Papillary Thyroid Carcinoma
TAM	Tumor-associated macrophages
TC	Thyroid Cancer
TCGA	The Cancer Genome Atlas
TME	Tumor microenvironment
THCA	Thyroid Carcinoma
