# Supplementary material for: TIMP1 and DPP4 Promote Tumor Progression by Regulating Lactate Metabolism in Papillary Thyroid Carcinoma
Source: Cancers (Basel). 2026 Apr 16;18(8):1264. doi: 10.3390/cancers18081264 (PMC13114815; doi:10.3390/cancers18081264)
Supplement: Supplementary file 1 [file cancers-18-01264-s001.zip › cancers-4225985-supplementary.pdf]

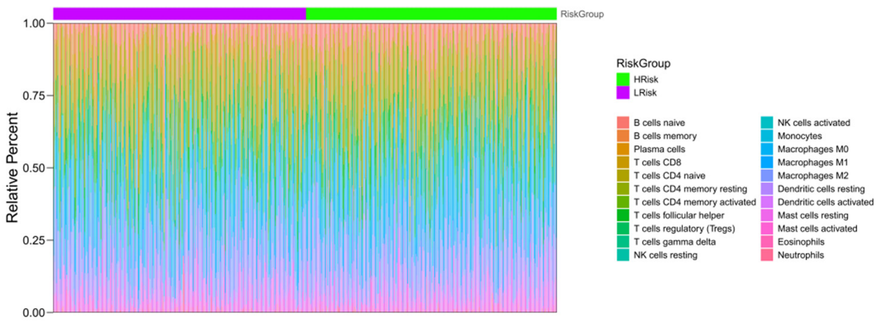

Figure S1. The distribution of immune levels of different immune factors in samples.

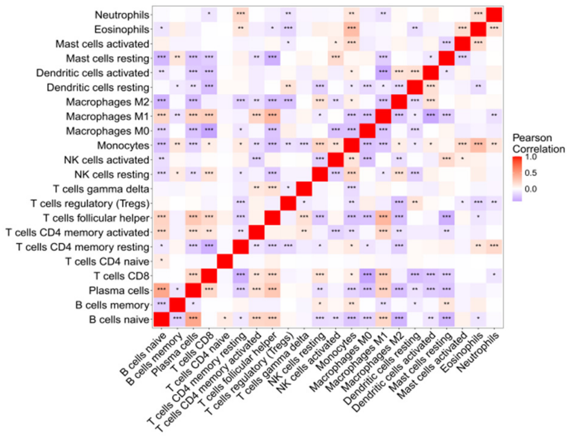

Figure S2. The interrelationship among immune factors on;$^{*}P<0.05$， $^{**}P<0.01$ ， $^{***}P<0.001$.

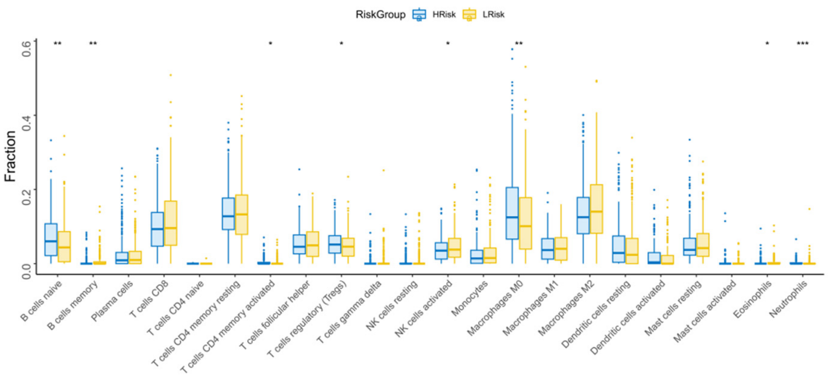

Figure S3. Immune factor levels in high-risk and low-risk groups;$^{*}P<0.05$， $^{**}P<0.01$ ， $^{***}P<0.001$.

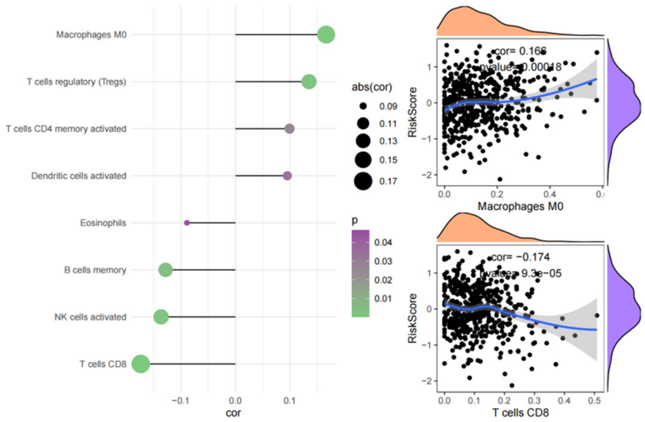

Figure S4. The relationship between risk score and immune cells;*P*<0.001.

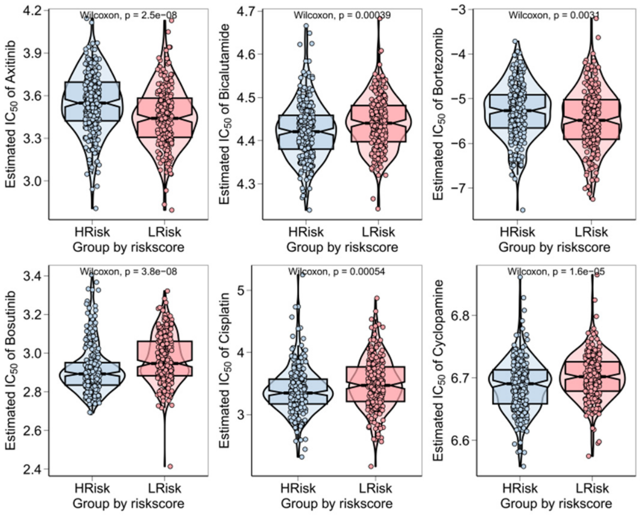

Figure S5. The level of risk assessment is significantly correlated with the patients' sensitivity to drugs such as Axitinib, Bicalutamide, Bortezomib, Bosutinib, Cisplatin and Cyclopamine.

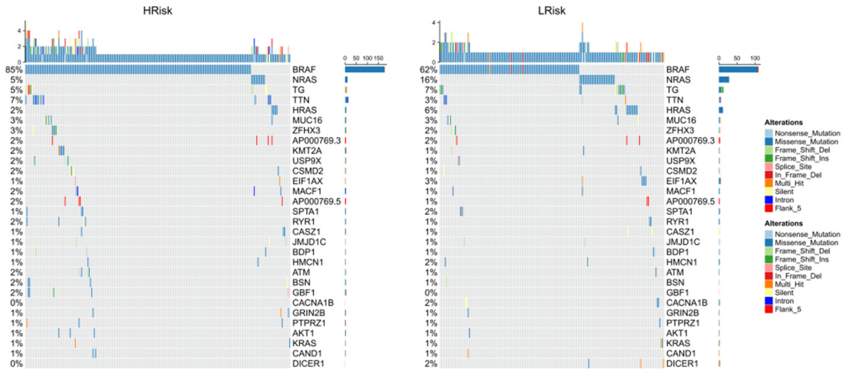

Figure S6. Distribution of mutation types in high-risk and low-risk groups results.

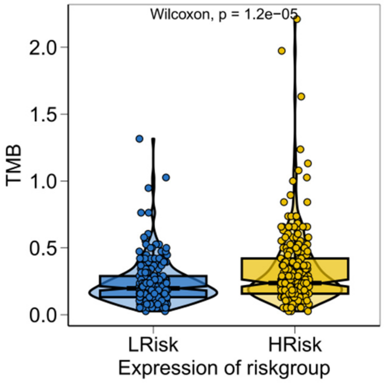

Figure S7. The results showing the difference in tumor mutational burden between the high-risk group and the low-risk group indicated that the tumor mutational burden in the high-risk group was lower than that in the low-risk group.

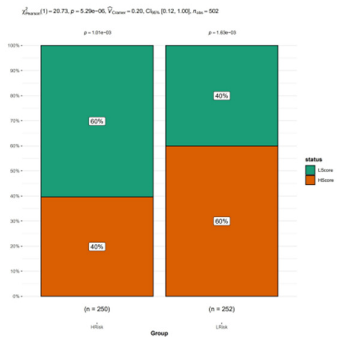

(a)

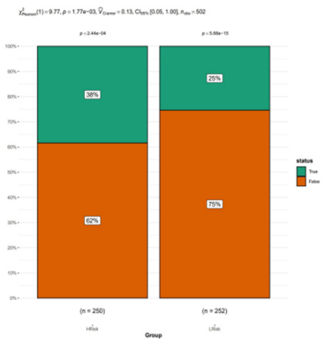

(b)

Figure S8. Differences in tumor immune function between high-risk and low-risk groups:There were significant differences in variables such as Responder and Exclusion between the high-risk and low-risk groups.
